# Aged Lignocellulose Fibers of Cedar Wood (9th and 12th Century): Structural Investigation Using FTIR-Deconvolution Spectroscopy, X-Ray Diffraction (XRD), Crystallinity Indices, and Morphological SEM Analyses †

**DOI:** 10.3390/polym16233334

**Published:** 2024-11-28

**Authors:** Yousra Bouramdane, Mustapha Haddad, Adil Mazar, Saadia Aît Lyazidi, Hicham Oudghiri Hassani, Abdellatif Boukir

**Affiliations:** 1 Laboratory of Microbial Biotechnology and Bioactive Molecules LBM2B, Faculty of Sciences and Techniques of Fez, Sidi Mohammed Ben Abdellah University, B.P. 2202, Imouzar Road, Fez 30007, Morocco; 2 Laboratory of Spectrometry of Materials and Archaeomaterials LASMAR, Faculty of Sciences, University Moulay Ismail, Meknes 50100, Morocco; m.haddad@umi.ac.ma (M.H.); s.aitlyazidi@umi.ac.ma (S.A.L.); 3 Institut Africain de Recherche en Agriculture Durable (ASARI) Laâyoune, University Mohammed 6 Polytechnic UM6P, Ben Guerir 43150, Morocco; adil.mazar@um6p.ma; 4 Laboratory of Engineering, Organometallic, Molecular Materials and Environment (LIMOME), Faculty of Sciences, Sidi Mohammed Ben Abdellah University, Fes 30000, Morocco; hicham.oudghirihassani@usmba.ac.ma

**Keywords:** cedar wood, lignocellulosic fibers (lignin, cellulose, hemicelloses), IR-spectroscopy/deconvolution, XRD, cellulose crystallinity index, SEM analyses, wood deterioration

## Abstract

The characterization of lignocellulosic biomass present in archaeological wood is crucial for understanding the degradation processes affecting wooden artifacts. The lignocellulosic fractions in both the external and internal parts of Moroccan archaeological cedar wood (9th, 12th, and 21st centuries) were characterized using infrared spectroscopy (FTIR-ATR deconvolution mode), X-ray diffraction (XRD), and SEM analysis. The XRD demonstrates a significant reduction in the crystallinity index of cellulose from recent to aging samples. This finding is corroborated by the FTIR analysis, which shows a significant reduction in the area profiles of the C-H crystalline cellulosic bands (1374, 1315, and 1265 cm^−1^) and C-O-C (1150–1000 cm^−1^). The alterations in the lignin fraction of aging samples (from the 9th and 12th centuries) were demonstrated by a reduction in the intensity of the bands at 1271 and 1232 cm^−1^ (C_ar-_O) and the formation of new compounds, such as quinones and/or diaryl carbonyl structures, within the 1700–1550 cm^−1^ range. The SEM images of cedar wood samples from the 9th and 12th centuries reveal voids, indicating that the entire cell wall component has been removed, a characteristic feature of simultaneous white rot fungi. In addition, horizontal “scratches” were noted, indicating possible bacterial activity.

## 1. Introduction

Archaeological wood, as defined by Florian, refers to “dead wood, used by an extinct human culture, modified or unmodified for or by use, and discarded in a specific natural environment” [1]. The condition of archaeological wood can range from nearly intact to deeply deteriorated material. However, age alone does not determine the extent of deterioration; it depends on factors such as the type of wood, environmental conditions, and the duration of exposure to aging processes. This underscores that wood deterioration varies significantly based on preservation conditions. 

Wood is mainly composed of three types of polymers: cellulose, hemicellulose, and lignin. These components are tightly intertwined and chemically linked through non-covalent forces and covalent bonds [2]. Cellulose consists of D-glucose monomer units that are cross-linked between molecular chains through numerous hydrogen bonds, which may result in the development of both amorphous and crystalline structures [3]. Hemicellulose is an amorphous biopolymer composed of branched heterogeneous heterocyclic sugars: pentose (C5) such as xylose and arabinose, as well as hexose (C6) such as glucose, mannose, galactose, and glucuronic acid in the branches. The branched heteropolymer constituents as well as the presence of esterified functional groups (-CO_2_CH_3_) make the hemicellulosic structure more sensitive to degradation and hydrolysis than the linear homogeneous cellulosic biopolymer (amorphous and/or crystalline polysaccharides) [3,4,5]. As for lignin, it is identified as being an amorphous biopolymer composed of phenolic nuclei to which radicals of the methoxy and hydroxyalkyl groups are connected to different positions of the aromatic ring forming the following most common monomers: syringyl alcohol, guaiacol, and sinapylic alcohol [3,6,7]. 

Attenuated total reflectance–Fourier transform infrared spectroscopy (ATR-FTIR), X-ray diffraction (XRD), and scanning electron microscopy (SEM) are among the more frequently employed tools to examine the chemical and morphostructural composition of wood [8,9,10,11], particularly cedar softwood [12,13,14]. FTIR spectroscopy has been widely utilized to examine the chemical transformations in wood resulting from weathering, chemical treatments, and biodegradation processes [5,6,9,15]. This technique is also applicable to archaeological wood samples, aiding in the assessment of suitable conservation methods. Moreover, numerous studies have explored FTIR approaches for analyzing wooden artifacts [15,16]. Additionally, the deconvolution of IR bands enhances the analysis by allowing integration of the areas of bands that exhibit changes over time. This process provides insights into the crystallinity index, which reflects the structural evolution of cellulose. The crystallinity index is calculated by examining the ratio of crystalline bands, offering a quantitative measure of the wood’s structural transformations [17,18]. In XRD analysis, the crystallinity index (CrI) is influenced not only by the proportion of crystalline material but also reflects variations in crystallite size [19]. XRD has long been employed to study the structural characteristics of cellulose and cellulose-based materials [20,21,22,23,24]. SEM images inform on the state of degradation of the cellulose fibers, which can be present as dramatic damage to the morphological surface, but can also be used to ascertain the state of conservation of the cellulose structure [25,26]. 

In this study, we examined two samples of archaeological cedar wood from the 9th and 12th centuries, sourced from historic mosques (Jamaa Al-Anouar and Jamaa Lakbir) in Fez and Meknes, Morocco, respectively, and compared them to a recent cedar wood sample from the 21st century. This comparison aimed to gain insights into the chemical composition, functional groups, structural features, and aging-induced transformations due to factors such as time, UV exposure, fungal biodeterioration, and natural weathering. Notably, we calculated the crystallinity index via FTIR spectroscopy—a method that, to our knowledge, has not previously been applied to Moroccan cedar woods to assess crystallinity changes in aged samples. By investigating these transformations quantitatively and linking them to structural stability, this study offers a transferable approach for understanding material degradation, valuable in both conservation and broader preservation science contexts.

## 2. Materials and Methods

### 2.1. Cedar Wood Samples

Two archeological cedar wood timbers dating from the 9th and 12th centuries were collected from two historical mosques located in the two imperial cities of Morocco (Fez and Meknes). The aging one was provided from the mosque of Jamaa AL Alanouar (Fez city), while the other one was taken from an old mosque of Jamaa Lakbir (Meknes city). The two archeological samples were compared to a recent one from the 21st century and derived from the forest of the Azrou region (Middle Atlas, Morocco). The samples were stored at ambient temperature.

The two external and internal faces related to each sample constituted a set of six samples (faces) to analyze by ATR-FTIR/deconvolution mode, X-ray diffraction, and SEM-EDX analysis.

The internal parts were sampled from the inner section of the decayed wood, specifically at a depth of 1 cm. Both samples have the following dimensions in the tangential, radial, and longitudinal directions: 1 × 2 × 3 cm. The attributes of the experimental materials are detailed in Table 1.

### 2.2. ATR-FTIR Analysis

FTIR has been applied to analyze the degradation levels in archaeological woods. FTIR spectra were obtained using a VERTEX 70—BRUKER (Billerica, MA, USA) spectrometer coupled to a Hyperion microscope, with the following parameters: 124 scans per sample, spectral resolution of 4 cm^−1^, and a wavenumber range from 4000 to 400 cm^−1^. A single diamond reflection attenuated total reflectance (ATR) accessory was utilized. Small rectangular pieces of the wood material samples were analyzed by scanning the total surface in order to obtain the best spectra based on our expectations. The spectra were normalized to 1375 cm^−1^; this band was chosen because it is characteristic of C-H bending in cellulose I and II and remains relatively stable, even in external samples. Normalizing the spectra with this band allows for a reliable comparison of peak intensities across samples by minimizing potential variations. XPS Peak Fit software was used for the deconvolution of some bands of the infrared spectrum with a mixture of Gaussian—Lorentzian functions without any correction in the baseline.

The crystallinity index was calculated using the following ratios: A_1430_/A_895_ (O’Connor et al., 1958) [17], A_1372_/A_2900_ (Nelson and O’Connor 1964) [27], and A_1375_/A_895_ (Oh et al., 2005) [23]. 

### 2.3. XRD Measurement

To investigate the crystallinity of cellulose in the wood samples, XRD measurements were conducted on an XPERT-PRO (Malvern Panalytical, Almelo, Netherlands) diffractometer using Cu-Kα radiation (λ = 1.5406 nm). The instrument was operated at 40 kV and 30 mA, with a scanning rate of 40 s per step and an angular step size of 0.017 degrees. The crystallinity degree was determined using Equation (1) proposed by Hermans and utilized by Popescu [28].

(1) $$\mathrm{CrI}\;(\%)=\frac{A_{cryst}}{A_{total}}\times 100$$

where CrI (%) is the crystallinity degree, A_cryst_ is the sum of the crystalline band areas, and A_total_ is the total area under the diffractogram.

### 2.4. SEM Analysis

The FEI Quanta 200 (FEI Company, Hillsboro, Oregon, USA) EDAX microscope was used for SEM analysis. The microphotographs were recorded at a high resolution (magnification ×1000). The micro-images were recorded in secondary and backscattered electron mode. The images of the degraded samples obtained by SEM analysis are blurred and difficult to use because of the accumulation of charges on the surface of the analyzed samples. To remedy these problems (charge effects) and to obtain clear images, it is recommended to perform a metallization treatment to make the samples conductive under the effect of electron beams and subsequently allow them to be observed by SEM. For this reason, the degraded samples were sputtered with EDWARDS Scancoat Six SEM coating to cover them with a thin layer of carbon (between 20 and 40 nm) while respecting their topography. The metalized samples are then made conductive under the effect of the SEM electron beam. Finally, the tested sample was loaded into the SEM chamber operating under high vacuum conditions.

### 2.5. Degradation Reactions of Lignocellulosic Materials

**Figure 1:**
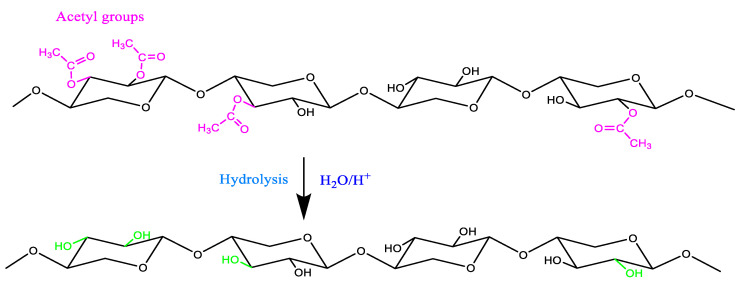
Hydrolysis reaction of acetyl groups in hemicelluloses.

**Figure 2:**
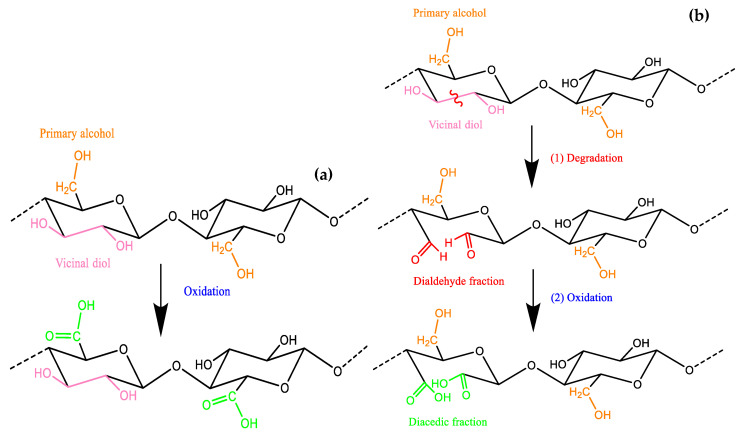
(**a**) Oxidation reaction of primary alcohol in cellulose. (**b**) Degradation reaction of vicinal diol and its oxidation to diacedic fraction in cellulose.

**Figure 3:**
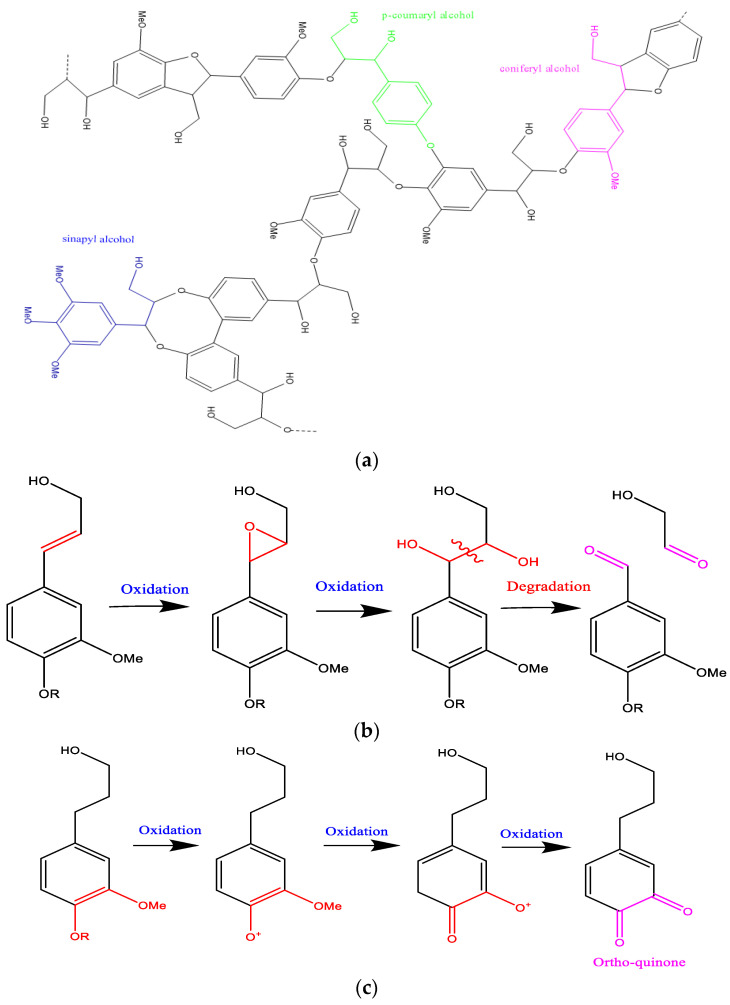
(**a**) Chemical structure of lignin. (**b**) Oxidation and degradation of lateral chain in lignin. (**c**) Oxidation of aromatic ring in lignin.

The degradation of wood components primarily results from chemical, biological, and environmental processes affecting the three major polymers: cellulose, hemicellulose, and lignin. The different degradation reactions that can occur have been illustrated in Figure 1 (hydrolysis of hemicellulose), Figure 2 (oxidation and degradation reactions of cellulose), and Figure 3 (oxidation of lignin).

## 3. Results

### 3.1. ATR-FTIR Characterization

**Figure 4:**
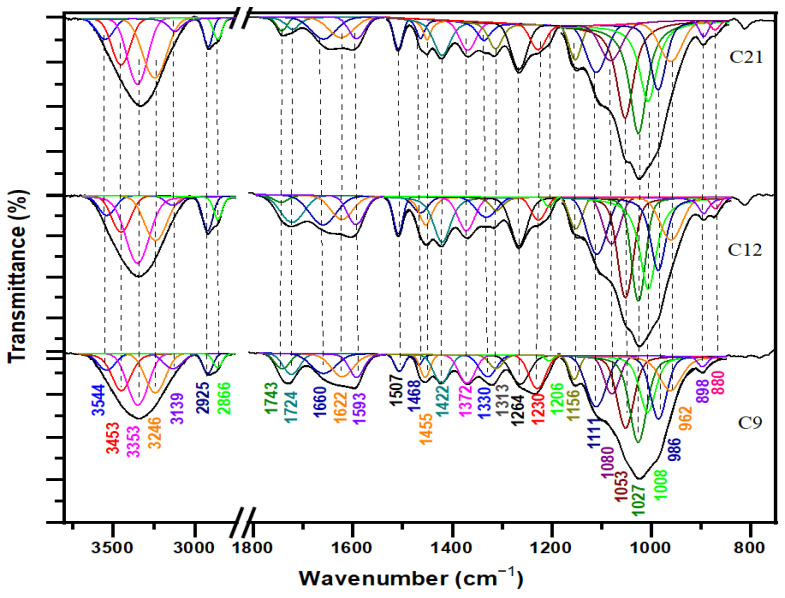
The IR spectra from bottom to top in black color represent the normal spectra with overlapping bands of three internal samples (C9: sample dating to 9th century, C12: sample dating to 12th century, and C21: sample dating to 21st century), while the other remaining colored spectra are recorded in deconvolution mode representing nonoverlapped and well-resolved bands.

**Figure 5:**
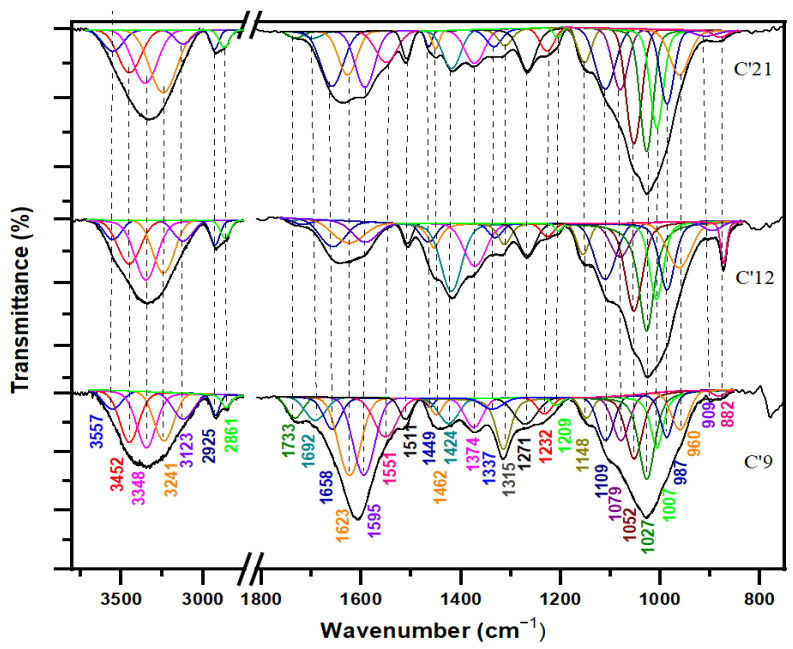
The IR spectra from bottom to top in black color represent the normal spectra with overlapping bands of three external samples (C’9: sample dating to 9th century, C’12: sample dating to 12th century, and C’21: sample dating to 21st century), while the other remaining colored spectra are recorded in deconvolution mode representing nonoverlapped and well-resolved bands.

**Figure 6:**
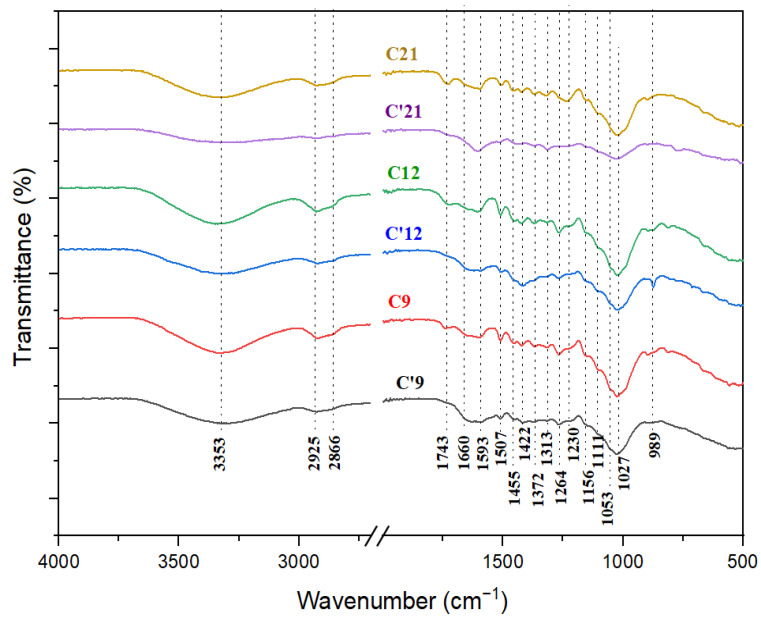
Superposition of FTIR spectra of both cedar wood samples: internal (C21: 21st century, C12: 12th century, C9: 9th century) and external (C’21: 21st century, C’12: 12th century, C’9: 9th century).

As reported in the literature data [3,29,30], the major bands related to the amounts of cellulose and hemicelluloses were found at 3348, 3241, 2925, 1733, 1462, 1424, 1374, 1315, 1156, 1111, 1027, and 898 cm^−1^, whereas the bands related to the lignin component were found at 1595, 1507, 1264, 1230, and 834 cm^−1^. At first glance, the broad peak at 3353 cm^−1^, along with the distinct peaks at 2866, 2925, and 1743 cm^−1^, indicates that cellulose, hemicellulose, and lignin are the major components in the wood samples. The FTIR spectra of the three internal samples are plotted in Figure 4, while the three external ones are illustrated in Figure 5. The FTIR superposition of the six samples (internal and external) is reported in Figure 6, and their assignments are detailed in Table 2, providing a clear comparison.

#### 3.1.1. Cellulose Fraction

The prominent peak at approximately 3353 cm^−1^ present in all samples is assigned to OH stretching vibration, which is characteristic of hydrogen bonding within the molecule [43]. Specifically, this vibration develops between the O(3)H-O(5) positions adjacent to the β-1,4 glycosidic bond of cellulose I [44,45]. The hydroxyl groups on the C(2), C(3), and C(6) carbons of cellulose are well known for their crucial role in forming inter- and intramolecular hydrogen bonds. These bonds greatly influence the physical properties of cellulose, such as its solubility, hydroxyl reactivity, and crystallinity, and are beneficial for the mechanical properties of the polymers, making them indispensable for various applications.

According to Noda [46], the α conformer of D-glucose in the cellulose fraction shows absorption bands at 1145, 1104, 1074, 1055, 1030, and 995 cm^−1^, while the β conformer is characterized by bands at 1158, 1108, 1080, 1035, 1020, and 992 cm^−1^. Our data (Figure 4 and Figure 5) reveal the β-anomeric conformation through the bands at 1156, 1111, and 1080 cm^−1^.

In the internal sample spectra of cellulose (Figure 4) from the recent sample (C21: 21st century) to the aging one (C9: 9th century), the C-H crystalline cellulose peaks (1313 and 1372 cm^−1^) did not exhibit any extensive modification. The profiles of 3700–3000 cm^−1^, 3000–2850 cm^−1^, and 1200–950 cm^−1^ showed relative observed variations that indicated weak structural alterations and a slight depletion in the cellulose structure. 

The spectral analysis of the external samples (Figure 5), comparing the recent cedar (C’21) to the aging wood (C’9), showed a shift of the band at 3452 cm^−1^ to a lower wavenumber and a slight reduction in band intensity. This change suggests that the cellulose in the archaeological wood has developed stronger hydrogen bonding interactions than those in the recent cedar softwood [15]. Additionally, the intensity of the bands from 2925 to 3452 cm^−1^ decreases significantly, indicating the deterioration of the crystalline fraction in cellulose. Furthermore, the crystalline cellulose bands (1315 and 1148 cm^−1^) underwent highly noticeable alterations, which were underlined by a gradual decrease in the intensity of these bands from the recent sample to the oldest ones (Figure 5: C’9 and C’12). Our results are in line with previous studies [15], confirming the observed patterns and properties.

The transition from recent to aging samples, both external and internal, shows a reduction in the intensity of the band at 1315 cm^−1^ (v_as_ CH_2_ in crystalline cellulose I) alongside an increase in the band at 1337 cm^−1^ (v_as_ CH_2_ in amorphous cellulose). According to Lionetto et al. [40], this suggests that the crystalline form transforms into amorphous during natural degradation.

The crystallinity indices obtained from the infrared data and the ratios used are presented in Table 3.

As shown in Table 3, the IR crystallinity indices decrease from the recent samples (C21 and C’21) to the aged ones (C12, C’12, C9, and C’9) and from the internal to the external ones, particularly those calculated from the ratio I_1372_/I_895_.

#### 3.1.2. Hemicellulose Fraction

The absorption peak at 1733 cm^−1^ in the spectra of the cedar softwood samples is due to the C=O stretching vibration, which originates from hemicelluloses (acetyl group) or the ester linkage of carboxylic groups found in ferulic and coumaric acids within lignin [37].

The relative absorbance of this peak has decreased from the recent cedar wood sample to the aged one, suggesting that most hemicelluloses and lignin may have been reduced or removed from the fibers due to natural degradation processes. The peak at 1648–1660 cm^−1^, attributed to the vibration of C=O of the ketone function, has decreased in relative intensity. The decrease in the relative intensity of the bands related to CH and CH_2_ deformation and the aromatic rings at 1449–1455, 1422–1424, 1372–1374, and 1313–1315 cm^−1^ [15,36] indicates an increase in the oxidation degree. Furthermore, the significant sharp peaks at 1148 and 1156 cm^−1^ establish the 1–3 bound xyloglucan fragments in the hemicelluloses [33].

Both the internal and external samples display a broad absorption band spanning 2250 to 3600 cm^−1^ in their infrared profiles (Figure 1 and Figure 2). This suggests the existence of bonded OH acidic groups (dimers), consistent with the corresponding C=O carboxylic signal observed at 1733–1743 cm^−1^, indicative of glucuronic acid in hemicelluloses [38,41]. The acidity was more pronounced in C21 (Figure 4) and C’21 (Figure 5) but was most noticeable in C’9 (Figure 5).

In the comparison between the recent sample (Figure 5: C’21) and the aging one (Figure 5: C’9) in the external spectra of hemicelluloses, there was a notable reduction in the intensity of the band at 1733 cm^−1^ (vC=O of hemicellulose) [29]. Concurrently, a new band emerged at 1692 cm^−1^, associated with aldehydes and conjugated carboxylic acid [35]. This new band suggests the formation of additional C-O and C=O bonds in the archaeological wood.

#### 3.1.3. Lignin Fraction

The 3400 cm^−1^ band, corresponding to hydrogen bonding, shows a progressive decrease in intensity from the recent sample (21st century) to the aging one (9th century), and from internal to external samples. This reduction is attributed to the degradation of lignin and the associated polar carbohydrate linkages [43]. 

Typically, in the spectra of aging cedar softwood, the bands associated with lignin (1593–1595, 1507–1511, and 1264–1271 cm^−1^, attributed to the C_ar_=C_ar_ stretching of the aromatic ring and C-O stretching in lignin, respectively) exhibit very low absorption intensities or may even be absent. The band at 1733 cm^−1^, assigned to C-O stretching in unconjugated ketones, is also noteworthy. This results from the low lignin content in this sample [29]. The progressive reduction and distinct variation in the intensity of the phenolic O-H band, the C_ar_=C_ar_ aromatic skeleton stretching mode (1600–1500 cm^−1^), and the aliphatic C-H (predominantly CH_3_) stretching vibration modes (3000–2850 cm^−1^) are indicative of the degradation occurring within the lignin matrix (Figure 5: C’21 and C’9). Additionally, the reduction in the intensity of the bands at 1264–1271 and 1230–1232 cm^−1^ (C_ar_=O) and the emergence of new compounds that might correspond to quinone nuclei and/or diaryl carbonyl structures within the 1700–1550 cm^−1^ range indicate structural deterioration (oxidation phenomena) within the lignin matrix [3,15,16,32,42].

The band at 1724–1733 cm^−1^ is assigned to the C=O stretching vibration of the unconjugated carbonyl group of ketone and the ester groups of acetyl (-C(=O)-OCH_3_). The 1264–1271 cm^−1^ band corresponds to the C-O stretching vibration in lignin, specifically linked to the C-O bond in guaiacol aromatic methoxy groups. This band exhibits higher intensity in the recent internal sample (C21) compared to the aging external sample (C’9). The peak at 1507–1511 cm^−1^ is associated with the stretching vibration (C_ar_=C_ar_) of the aromatic rings in lignin [29]. This band is largely absent in aging cedar wood samples. The band at 1593–1595 cm^−1^ is linked to the stretching vibration of syringyl units (aromatic ring), while the band at 1148–1156 cm^−1^ corresponds to the CH in-plane deformation typical of guaiacol units [29,42]. The vibrations of aromatic rings, indicated by the peaks at 1593–1595, 1507–1511, and 1422–1424 cm^−1^, suggest that delignification has occurred due to natural degradation in the aging sample (C’9) [15].

Cedar wood belongs to the division of pinophyta and family of pinaceae and is considered a coniferous softwood. The prominent round and flattened C_ar_-O bands with polar characteristics absorbing at 1230 and 1264 cm^−1^ (predominant peak) clearly indicate the presence of a guaiacol nucleus as a major constituent of the lignin matrix that is correlated to cedar softwood [47,48]. 

In the IR profile of the internal cedar wood, from the recent sample (Figure 4: C21) to the aging one (Figure 4: C9), various structural changes in the wood mainly influenced the broad signal in the 1700–800 cm^−1^ range. The decrease in intensity in the 1700–1550 cm^−1^ region indicates a reduction in the contribution of adsorbed water molecules (1630–1610 cm^−1^) [47,48]. 

The observed relative degradation of lignocellulosic components in internal cedar wood may be attributed in part to moisture and fungal activity, as reported by Moulay et al. [49] in their study on aging Moroccan cedar wood used in artworks.

Concerning the IR profiles of the lignin external samples (Figure 5), the majority of the IR bands within the range of 1700–800 cm^−1^ showed a reduction in their intensities, supporting a rise in the intensity of the 1700–1550 cm^−1^ bands corresponding to the oxidative region. These changes prove the structural deterioration of the lignin matrix. From the recent cedar softwood sample (Figure 5: C’21) to the aging one (Figure 5: C’9), the observed decrease in the intensity of the bands at 1595 cm^−1^ (C_ar_=C_ar_) and 1658 cm^−1^ (C=O) indicates a reduction in the lignin content (aromatic character and oxidized form: deconjugated carbonyl fractions), highlighting the severe and intensified deterioration of the structural lignin matrix. These findings are consistent with those reported in previous literature [3,15]. 

### 3.2. X-Ray Diffraction Measurement

**Figure 7:**
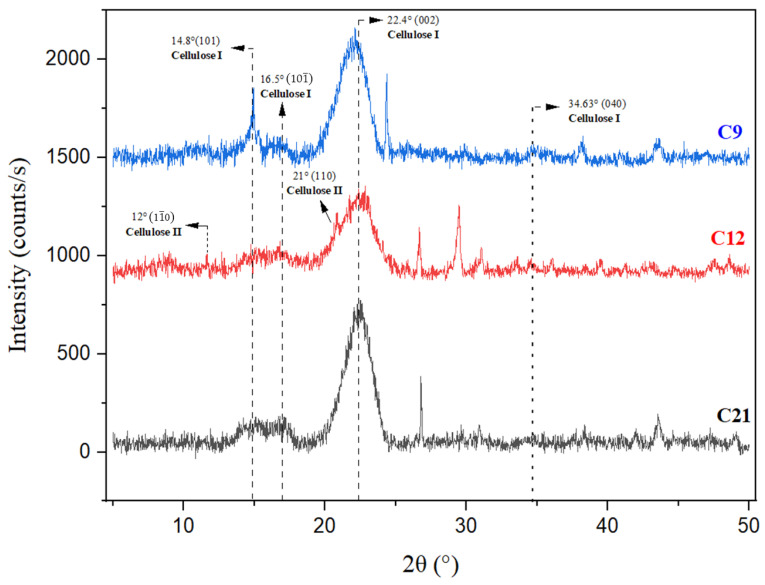
XRD diffractogram of three superposed internal cedar wood samples (**C9**: sample dating to 9th century, **C12**: sample dating to 12th century, **C21**: sample dating to 21st century).

**Figure 8:**
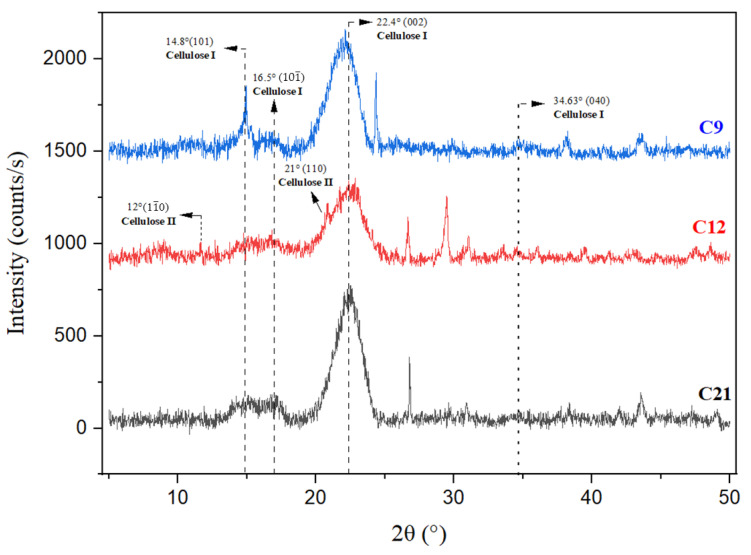
XRD diffractogram of three superposed external cedar wood samples (**C’9**: sample dating to 9th century, **C’12**: sample dating to 12th century, **C’21**: sample dating to 21st century).

The cedar softwood samples underwent X-ray diffraction analysis to examine the changes induced by natural degradation, study the evolution of polymorphs, and measure their crystallinity content. The XRD diffractograms of the three internal samples are presented in Figure 7, while those of the external samples are depicted in Figure 8. The crystallinity index (CrI) values for all six samples are summarized in Table 4.

The cedar wood samples showed comparable diffraction patterns, which can be decomposed into three primary peaks at 2θ~14.8°, 2θ~16.9°, and 2θ~22.4° along with a lower peak at 34.63° 2θ characteristic of cellulose $I_{\beta }$ [3,50,51] and the references cited therein. These diffractions were attributed to the crystallographic planes (101), (10$\bar{1}$), (002), and (040), respectively [51,52,53,54,55,56].

The primary peaks of cellulose I are observable in all spectra, suggesting that the crystal structure of cellulose has remained unchanged throughout the natural degradation process.

In the internal sample from the 12th century, the two peaks observed at approximately 12° and 20.7° suggest the presence of cellulose II. According to Carillo-Varella et al. (2018, 2019) [50,57], the peaks attributed to cellulose II appear at 2θ~12°, 2θ~20°, and 2θ~20.7°. These diffractions correspond to the crystallographic planes (1$\bar{1}$0), (110), and (020), respectively.

According to Klemm et al. (2005) [58], in the two forms of cellulose, I and II, the distribution of hydrogen bonds differs slightly. In cellulose I, which is in a metastable state, the glucopyranose chains are arranged in a parallel orientation, whereas in cellulose II, which is thermodynamically stable, they are oriented in an antiparallel manner. 

The amorphous phase of cellulose is characterized by a very large signal in the form of a hump located in the 2θ range from ~18° to 19°. The presence of this hump could also be correlated to the contribution of the amorphous fractions of lignin and xylan (hemicellulose) by forming a diffuse scattering halo in the 2θ range from ~12° to 27°, ‘overlapping the crystalline diffraction peak of cellulose’ [3,59]. All the XRD diffractograms of the external parts (Figure 5) of cedar softwood displayed a shoulder on the (200) peak, appearing at approximately 21 2θ angles, which is attributed to the (102) crystallographic plane of cellulose I_β_ [60]. This peak is not consistently present in all type I cellulose samples [61]. Concerning the aging samples, a significant decrease in the intensities was observed from C’12 to C’9. 

According to Poulain et al. (2019) [62], shifting a peak is generally a result of a change in the chemical composition or distortion of the crystallite. However, the variations in intensity are most probably the result of compositional changes in the unit cell or changes in the crystallite order. In the XRD patterns, the appearance of broad peaks instead of sharp ones may be attributed to several factors. These include the presence of small crystallite sizes, which broaden peaks, or defects in the crystal lattice. These factors can lead to reduced material organization, contributing to the broadening of peaks and increased diffusion in crystalline patterns [18]. 

In the external samples, the reduction in intensity of the peak at 22.4° (cellulose I) and the emergence of a new, very weak peak around 2θ of 12°, along with a weak peak at 20°, indicate a transition or contribution to the content of cellulose II. This result corroborates well with the literature data of French et al. (2013) [62], Hajji et al. (2016) [39], Carillo-Varela et al. (2018) [50], and Boukir et al. (2019) [3].

As seen in Figure 8, the crystalline peaks decreased in intensity from the recent sample (C’12) to the aging one (C’9), indicating the decomposition of a crystalline form [63]. The crystalline and amorphous fractions of cellulose underwent structural and chemical modifications. Amorphous cellulose is typically more reactive and vulnerable to breakdown than crystalline cellulose, which is stronger and less reactive [13].

According to Nelson and O’Connor (1964) [27], such a change in molecular conformation might depend on or coincide with a rearrangement of intramolecular hydrogen bonds. The intermolecular hydrogen bonding system must be significantly disrupted when the native cellulose I lattice is destroyed. These processes likely provide enough energy to also disturb the intramolecular system. This would allow the rotation of anhydroglucose units around the glycosidic linkage into positions favorable for forming a new set of intramolecular hydrogen bonds. Such a rearrangement of intramolecular bonds would tend to stabilize the new conformation, whether amorphous or in the form of cellulose II.

The development of supplementary diffraction peaks from 2θ~37° to 2θ~50° suggests the presence of extraneous components within the wood composition. These may result from residual fractions remaining after the decomposition of wood components and/or the production of chemicals by living organisms.

The crystalline and the amorphous fraction of cellulose are two interconnected phases. The crystalline arrangement of cellulose molecules shows a degree of uniformity, characteristic of the monoclinic crystal system. The amorphous phase exists between these crystalline regions, with a gradual transition and no distinct separation. Under specific conditions, wood degradation leads to the transformation of the crystalline cellulose phase into an amorphous phase [7].

The CrI results show that all samples present low crystallinity indices (below 41%), particularly for the oldest ones (14% for C’9 (9th century)). It appears that the samples experienced significant deterioration and/or that the type of wood studied has a structure particularly susceptible to degradation.

For internal samples, the measured crystallinity index fell in the following order: C21 > C12 > C9 (Table 4), while for samples that had been subjected to degradation, the index declined in the following order: C’21 > C’12 > C’9 (Table 4). In accordance with this outcome, from the most recent sample (C21) to the aging one (C9), as well as from the least external sample (C’21) to the most aging one (C’9), the crystallinity of wood samples declines. This indicates that the native state of wood cellulose was initially crystalline but transitioned to an amorphous state upon exposure to the natural degradation process.

### 3.3. SEM Characterization

**Figure 9:**
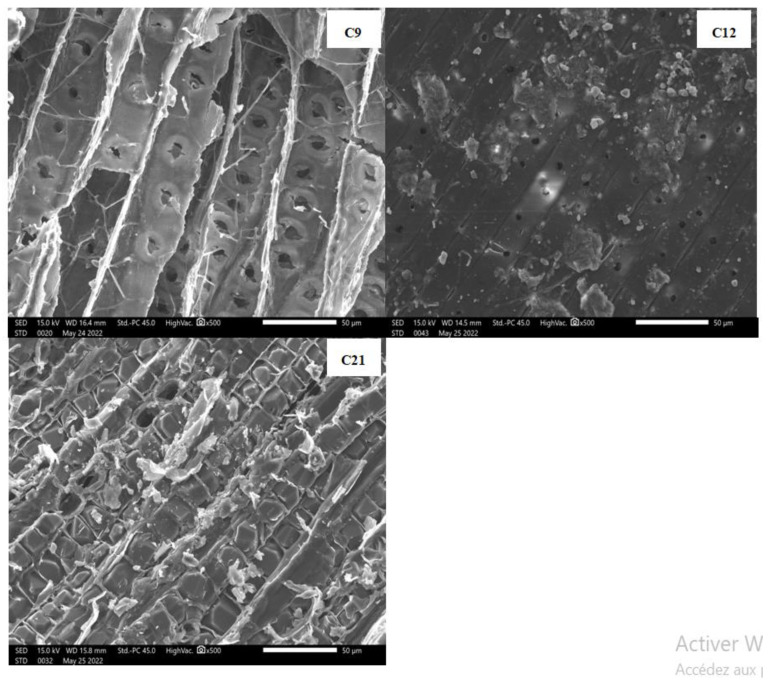
SEM micrographs of three internal cedar wood samples (**C9**: sample dating to 9th century, **C12**: sample dating to 12th century, and **C21**: sample dating to 21st century).

**Figure 10:**
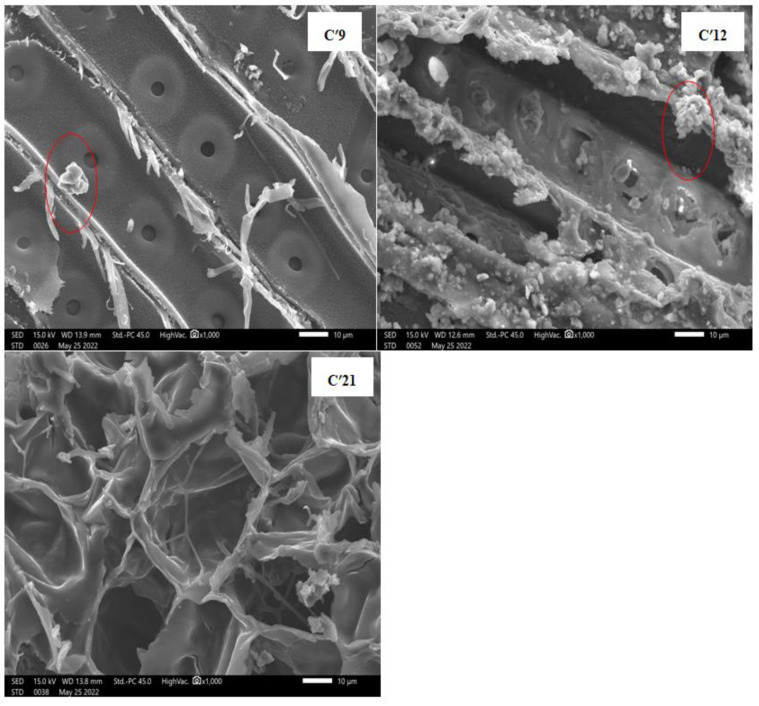
SEM micrographs of three external cedar wood samples (**C’9**: sample dating to 9th century, **C’12**: sample dating to 12th century, and **C’21**: sample dating to 21st century).

Various types of degradation impact archaeological and historical wood differently, resulting in distinct changes in its anatomical structure. These morphological degradation patterns are instrumental in identifying the type of degradation and the responsible agent. SEM analysis is employed to monitor and elucidate these changes, revealing crucial insights. Figure 9 and Figure 10 display the SEM images of all wood samples.

#### 3.3.1. Non-Degraded Parts

Lignocellulosic materials exhibit adhesive properties, illustrated in Figure 9: C21, where wood chips form a bundle-like structure. Many fibers are tightly bound, characterized by large fiber diameters and smooth surfaces [7]. In Figure 9, the SEM images reveal structures in the form of filaments and spheres that are related to fungal hyphae and spores.

The SEM morpho-structural analyses of the internal samples (Figure 9: C21, C12, and C9) reveal a fibrous structure composed of vascular bundles with varying sections and a heterogeneous distribution of fiber dimensions, as well as the presence of numerous voids due to the high porosity level. In the aging samples C9 and C12, the bordered and non-bordered vessels shown in the SEM images (Figure 9) have different diameter values ranging from 4 to 6 µm. However, the bordered and non-bordered vessels were absent in the most recent sample (Figure 9: C21). The morphological examination of the recent internal sample (Figure 9: C21) shows the presence of several agglomerates of crystalline cellulose that are clearly visible with longitudinal rows and regular distribution, arranged into near micro-fibrils and orientated in the same direction, which offers information on the cellulose rearrangement. The obtained data show that a significant amount of glycosidic ring cellulose is present in the compact microstructure, allowing for the establishment of molecular hydrogen bonds (inter and intra) and thus conferring stability to the microstructure, indicating the almost complete absence of crystalline degradation. The results of the XRD (CrI = 40.9%) and FTIR analyses are in the previous section.

The alteration is clearly visible in the SEM images of the aging samples (Figure 9: C9 and C12), which show a lot of damage in the wood structure related to the sample’s metallization preparation step that may be partially correlated to the contribution of several fungi but is not related to the phenomenon of the natural degradation effect. 

#### 3.3.2. Degraded Parts

The rate of wood decay can indeed be influenced by a combination of factors including fungi, wood nutritional content, water availability, oxygen levels, humidity, temperature, and density. Certain fungi specialize in the degradation of specific types of wood [62].

The degradation is visible and clearly supported by the changes in the structure of aging cellulose fibers, which are more affected and prominent in the case of the external sample (Figure 10: C’9 and C’12), informing us about the behavior of the cell wall and cellulose fibers after a large break, which affected the microstructure. The impact of the alteration is clearly visible and may be explained by the following effects: prolonged exposure and environmental weathering conditions. From the recent sample (Figure 6: C’21) to the aging one (Figure 10: C’9), a gradual and slow degradation of the wood structure was observed, characterized by the gradual separation of fibers and microfibril bundles from the surface. Additionally, there was an increase in the size of bordered pits and other pores.

Both images of the aging wood (Figure 10: C’9 and C’12) show deterioration of bordered pits resulting from weathering, in addition to the foreign deposits on the wood surface. The diameter of these bordered pits ranges from 8 µm (C’12) to 8 nm (C’9). Furthermore, the SEM images (shown in Figure 10: C’9 and C’12 as a white ellipse) reveal the presence of fungi (micro-organism colony), which is demonstrated to partially contribute to the damage caused but is less affected in C’21 than in C’12 and C’9. This phenomenon is due, in part, to hydrogen bonds reinforcing a large number of crystalline domains, conferring some stability to the cellulose fibers that comprise the compact microstructure.

In the SEM image of the aging and external samples (Figure 10: C’9 and C’12), the depletion of promoting agents suggests a strong depolymerization of cellulose involving both amorphous and crystalline forms (CrI (C’9) = 14.53% and CrI (C’12) = 33.3%), which is more noticeable in the amorphous phase and may be related to its sensitivity to hydrolysis reactions, UV sunlight, humidity, and other natural environment factors.

Determining the exact diameters and vessels of the fibers in the aging external samples (Figure 10: C’12 and C’9) appears to be more difficult, owing to the deformation of the structure of the fibers under the influence of mechanical forces and/or the presence of a significant number of altered fibers with different diameters. In Figure 10, voids were observed in both samples C’9 and C’12, suggesting the complete removal of cell wall components, a characteristic often associated with simultaneous white rot fungi [64].

Horizontal “scratches”, along with other observed changes, suggest possible bacterial activity [65]. In natural conditions, both mold fungi and wood-destroying fungi degrade the carbon-rich components of wood to form large fruiting bodies that release extensive amounts of spores. This process is visible in the images of the external and internal aging samples (Figure 9: C9 and C12) (Figure 10: C’9 and C’12) [10,11]. The strategy employed by mold fungi appears to involve creating a dense network of hyphae on the wood’s surface while secreting enzymes and organic acids into the wood’s interior [66]. 

The SEM images of recent samples (Figure 9: C21 and Figure 10: C’21: 21st century) appear intact and show very close fibrils randomly distributed with irregular porosity. The cell wall structure is highly disordered, and the fibers are in the beginning stages of formation, undergoing a metamorphosis phase, conferring some fragility to the youngest cedar wood.

The appearance of the less abundant and more distant fibers in the aging samples (Figure 9 and Figure 10: C9, C’9, C12, and C’12), with thin and less regular rays, showing a disorganized distribution, suggests the presence of a lower amount of glycosidic OH bonded due to the loss of sugar molecule fragments responsible for the alteration, conferring a lower degree of inter- and intra-molecular hydrogen bonding confirmed by the FTIR spectrum and XRD results (see previous sections), leading to a very weak cohesion and stability in the microstructure. Depolymerization of cellulose fibers occurs as a result [42,66], with a quasi-absence of crystalline forms supported by the XRD data (CrI (C’9) = 14.5% and CrI (C’12) = 33.3%). This leads to a significant and advanced degradation in the heterocyclic sugar rings affecting the fiber and aiding in the destruction of the microfibril cellulose structure. Several studies have explored the impact of aging on the structure of archaeological wood [34,67,68]. These studies discovered that decayed wood has a deteriorated ultrastructure, particularly in the area of the middle lamella. Furthermore, a distinct separation between the lamella in the secondary wall may occur. Nevertheless, the microfibrils remain unaffected, and the essential properties of the wood do not seem to experience substantial alterations.

According to Tomak et al. (2014) [69], prolonged weathering exposure tends to increase the surface roughness of samples, although this effect was not statistically significant for certain wood species. Additionally, moisture gradients between the surface and interior cause wood to swell and shrink, creating internal stresses. These uneven stresses, typically most pronounced near the surface, can lead to warping and face-checking.

Scanning electron microscopy (SEM) is considered an essential tool for examining the morphological characteristics of degraded wood at the cell wall level, assessing its damage, and identifying the causal agents of decay patterns.

## 4. Conclusions

ATR-FTIR spectroscopy and X-ray diffraction allow for observing the differences that appear in the structure of components between recent and aging cedar softwood samples. In the external samples, the peak intensities of the cellulose, hemicelluloses, and lignin components were significantly reduced, accompanied by the transformation of the crystalline form of cellulose to the amorphous one during natural degradation, which was confirmed by the CrI results. The CrI values obtained using both the Segal and O’Connor methods reveal a decrease in crystallinity from the recent sample to the aged one, as well as from the internal parts to the external ones.

The SEM images reveal the presence of fungus (microorganism colony) and the deterioration of bordered pits in the external parts of aging samples. 

The data obtained in this study show that the three analytical techniques (ATR-FTIR, XRD, and SEM) provide valuable insights into the structural and morphological properties of wood, as well as its degradation state. These methods lay a solid foundation for expertise in wood conservation and restoration, especially for preserving wooden artifacts.

## Figures and Tables

**Table 1:** Description of six cedar wood samples proposed for analyses and their relative age (3 internal faces: C9, C12, and C21; and 3 corresponding external faces: C’9, C’12, and C’21).

Centuries	Parts of Wood	Abbreviations
9th	Internal face	C9
	External face	C’9
12th	Internal face	C12
	External face	C’12
21st	Internal face	C21
	External face	C’21

**Table 2:** FTIR band attributions in the cedar wood (internal and external samples).

Wavenumbers (cm^−1^)		Band Ascriptions	References
Non-Degraded Samples	Degraded Samples		
3600–3000	3600–3000	vOH: Lignocellulose (intra- + inter-molecular hydrogen bond)	[29]
3246	3241	vOH: allomorph cellulose I_β_ (O6-H6….O3: intermolecular hydrogen bonds in carbohydrates)	[3,15,31]
3000–2850	3000–2850	vC-H aliphatic: CH_2_ + CH_3_ + CH in holocellulose + lignin	[7,32]
2925	2925	v_as+s_ CH_2_ + v_as+s_ CH_3_: holocellulose + lignin	[3,32,33]
1743	1733	vC=O of hemicellulose: unconjugated carbonyl of acetyl and carboxyl and/or glucoronate; vC=O esterified phenolic acid in lignin and/or ferulate	[3,29,34]
1724	-	vC=O of aldehyde	[35]
-	1692	vC=O of conjugated carboxyl acid	[35]
1660–1621	1660–1621	vC=O of diconjugated carbonyl ketone in lignin: quinone, or *p*-quinone, Ar-C(=O)-Ar, Ar-C(=O)-C=C, C=C-C(=O)-C=C	[3,32,36]
1622	1623	OH bending of adsorbed water	[9]
1595–1507	1595–1507	vC_ar_=C_ar_ of lignin	[3,29,32,37]
1468	1462	δ_as_ CH_3_ of acetyl pyranose in hemicellulose; δ_as_ CH_3_ of methoxyl in lignin; δ_as_ CH_2_ of cellulose; δ_as_ CH_2_ in lignin and carbohydrates	[3,15,33]
1455	1449	δCH_2_ in lignin + δO-H of cellulose	[15]
1422	1424	δCH_2_: scissoring in crystalline cellulose; lignin; hemicellulose + lignin	[3,38]
1372	1374	δ_as_ CH from –OCH_3_; O-H and C-O of phenol and tertiary alcohol; C-H bending in cellulose I and II	[3,34,39]
1330	1337	δCH of methyl groups in methoxy in amorphous cellulose; δC_1_-O in syringyl derivative	[15,37]
1313	1315	δCH_2_ of crystalline cellulose: wagging; δCH_2_: rocking	[3,34,39]
1264	1271	δC_ar_-O: guaiacyl skeleton in lignin + δC-O of cellulose	[3,35,40]
1230	1232	δC_ar_-O syringyl skeleton in lignin + δC-O-C ester groups in hemicellulose (xyloglucan)/lignin + δC-O carboxyl group; δO-H of carbohydrate and COOH	[3,29,33,35]
1156	1148	δ_as_C-O-C: bridge of -(1-4)-glycosidic in crystalline cellulose; δC-O of glucopyranose; δC-O-C of carbohydrate; δC-H of carbohydrate	[29,33]
1111	1109	δC-O: alcohol in carbohydrate, δC-O side chain lignin	[41,42]
1053	1052	δC–O stretching of secondary alcohols	[35]
1027–1007	1027–1007	δC-O: primary alcohol of carbohydrate; C-O- ether of cellulose or CH_3_-O in lignin; CH_3_-O- of ester and -O-4 linkages in lignin	[15,33]
986	987	δCO in cellulose	[35]
898	882	δC-O-C of -(1-4)-glycosidic linkage: amorphous or crystalline; δC-H: rocking of -glycosidic bonds; δC-H holocellulose; δ_oop_C-H_ar_: deformation mode in lignin	[34,39,40]

The following acronyms are used: v stretching vibration mode; v_as_: asymmetric stretching vibration; v_s_: symmetric stretching vibration; v_as_ asymmetric bending vibration; δ_s_ symmetric bending vibration; δ: in-plane deformation mode; δ_oop_: out-of-plane bending vibration.

**Table 3:** IR crystallinity index values (CrI %): internal and external wood samples.

Sample Reference	A_1430_/A_909_ O’Connor et al. (1958) [17]	A_1372_/A_2900_ Nelson and O’Connor (1964) [27]	A_1372_/_A895_ Oh et al. (2005) [23]
C21	0.93	1.05	1.08
C’21	0.94	0.99	0.99
C12	0.89	1.07	1.05
C’12	0.86	0.98	0.96
C9	0.93	1.07	1.02
C’9	0.91	1	0.92

**Table 4:** DRX crystallinity index values (CrI %) and the percentage change: internal and external wood samples.

Sample Age	Crystallinity Index CrI (%)		% Reduction in CrI
	Internal	External	
**21st century**	C21: 40.9	C’21: 37.18	3.72
**12th century**	C12: 36.9	C’12: 33.3	3.6
**9th century**	C9: 36.4	C’9: 14.53	21.87

## Data Availability

The original contributions presented in the study are included in the article, further inquiries can be directed to the corresponding authors.
